# Contrasting responses of soil microbial biomass and extracellular enzyme activity along an elevation gradient on the eastern Qinghai-Tibetan Plateau

**DOI:** 10.3389/fmicb.2023.974316

**Published:** 2023-01-18

**Authors:** Shun Liu, Gexi Xu, Huanhuan Chen, Miaomiao Zhang, Xiangwen Cao, Miao Chen, Jian Chen, Qiuhong Feng, Zuomin Shi

**Affiliations:** ^1^Key Laboratory of Forest Ecology and Environment of National Forestry and Grassland Administration, Ecology and Nature Conservation Institute, Chinese Academy of Forestry, Beijing, China; ^2^Miyaluo Research Station of Alpine Forest Ecosystem, Lixian County, China; ^3^Ecological Restoration and Conservation on Forest and Wetland Key Laboratory of Sichuan Province, Sichuan Wolong Forest Ecosystem Research Station, Sichuan Academy of Forestry, Chengdu, China; ^4^Co-Innovation Center for Sustainable Forestry in Southern China, Nanjing Forestry University, Nanjing, China; ^5^Institute for Sustainable Plant Protection, National Research Council of Italy, Turino, Italy

**Keywords:** phospholipid fatty acids, soil microbial community, extracellular enzyme, microbial metabolism, soil P, elevation

## Abstract

Soil microbial community composition and extracellular enzyme activity are two main drivers of biogeochemical cycling. Knowledge about their elevational patterns is of great importance for predicting ecosystem functioning in response to climate change. Nevertheless, there is no consensus on how soil microbial community composition and extracellular enzyme activity vary with elevation, and little is known about their elevational variations on the eastern Qinghai-Tibetan Plateau, a region sensitive to global change. We therefore investigated the soil microbial community composition using phospholipid fatty acids (PLFAs) analysis, and enzyme activities at 2,820 m (coniferous and broadleaved mixed forest), 3,160 m (dark coniferous forest), 3,420 m (alpine dwarf forest), and 4,280 m (alpine shrubland) above sea level. Our results showed that soil microbial community composition and extracellular enzyme activities changed significantly along the elevational gradient. Biomass of total microbes, bacteria, and arbuscular mycorrhizal fungi at the highest elevation were the significantly lowest among the four elevations. In contrast, extracellular enzyme activities involved in carbon (C)-, nitrogen (N)-, and phosphorus (P)- acquiring exhibited the maximum values at the highest elevation. Total nutrients and available nutrients, especially P availability jointly explained the elevational pattern of soil microbial community, while the elevational variation of extracellular enzyme activities was dependent on total nutrients. Microbial metabolism was mainly C- and P-limited with an increasing C limitation but a decreasing P limitation along the elevational gradient, which was related significantly to mean annual temperature and total P. These results indicated a vital role of soil P in driving the elevational patterns of soil microbial community and metabolism. Overall, the study highlighted the contrasting responses of soil microbial biomass and extracellular enzyme activities to elevation, possibly suggesting the differences in adaption strategy between population growth and resource acquisition responding to elevation. The results provide essential information for understanding and predicting the response of belowground community and function to climate change on the eastern Qinghai-Tibetan Plateau.

## Introduction

Soil microorganisms are the active component in soil and play an outsized role in driving soil organic matter (SOM) decomposition, mineralization as well as nutrient immobilization due to their important contribution to soil living biomass (He Q. et al., 2020; Fan et al., 2021; Sokol et al., 2022). Soil microbial community composition is an important factor affecting microbial function besides microbial biomass (Whitaker et al., 2014). Bacteria prefer labile substrates, while fungi are prone to decomposing complex substrates (Hackl et al., 2005; Djukic et al., 2010). Their ability for decomposing SOM can be assessed by multiple extracellular enzyme activities that mediate the rate-limiting step in degrading complex compounds (Ivashchenko et al., 2021; Zuo et al., 2021). Soil microorganisms would invest resources to synthesize and secrete extracellular enzymes for the required nutrients (Sinsabaugh et al., 2013; Malik et al., 2020; Shao et al., 2021), potentially indicating the microbial demand for nutrients and microbial activity (Wu et al., 2021; Zhang et al., 2022). Many studies have focused on soil microbial community and extracellular enzyme activities involved in carbon (C), nitrogen (N), and phosphorus (P) cycles (Nottingham et al., 2015; Pollierer et al., 2015; Xu et al., 2017; Klimek et al., 2020; Ren et al., 2021). That is because tiny changes in C and N fluxes between soil and atmosphere may provide substantial feedback to climate change (Liu et al., 2020). Moreover, N and P are common nutrient-limiting factors for C sequestration in ecosystems (Elser et al., 2007).

Climatic conditions are regarded as important factors regulating soil microbial community and extracellular enzyme activities. However, studies assessing the responses of soil microbial community and metabolism to climate change remain contentious (Waldrop and Firestone, 2006). This suggests diverse effects of climate change (Ren et al., 2021). For instance, some studies note that microbial biomass and community composition are highly sensitive to environmental changes (Rousk and Bååth, 2011), whereas other studies found only minor shifts in microbial biomass and community composition through warming experiments (Streit et al., 2014). It needs to strengthen the study of soil microbial community and extracellular enzyme activity under varying climatic conditions, which can provide insight into soil biogeochemical processes and predict the response and feedback of soil function to climate change (Zhang et al., 2022).

Elevational gradients have been regarded as a powerful “natural laboratory” to investigate the response and feedback of soil function to climate change (Cui et al., 2021; Feng et al., 2021), particularly to detect the elevational patterns of soil microbial community and extracellular enzyme activity since the climate and biotic attributes change drastically within small spatial scales (Djukic et al., 2010; Siles et al., 2017; Klimek et al., 2020). Research on the elevational patterns of soil microbial community and extracellular enzyme activity has been extensive (Whitaker et al., 2014; Xu et al., 2014; He X. et al., 2020; Cui et al., 2021; Fan et al., 2021; Ivashchenko et al., 2021), but a consensus remains elusive. For example, Djukic et al. (2010) found that total microbial biomass was lower at mid-elevation, and fungi/bacteria ratio decreased with increasing elevation in European Alps. Conversely, Xu et al. (2015) and Liu et al. (2019) documented that biomass of total and functional groups generally peaked at the mid-elevation, and fungi/bacteria ratio increased with increasing elevation in Changbai Mountain of China. Furthermore, other studies also reported either an increasing, a decreasing or no clear trend in microbial biomass with elevation (Whitaker et al., 2014; Chang et al., 2016; Lei et al., 2017; Klimek et al., 2020). The lack of consensus possibly indicates that the elevational pattern of microbial biomass is site dependent, confirming the result of a global synthesis study (He X. et al., 2020). Similarly, the responses of extracellular enzyme activity to elevation are still ambiguous (Bayranvand et al., 2021; Ivashchenko et al., 2021; Liao et al., 2021).

The inconsistent results among studies on elevational patterns of microbial community and extracellular enzyme activity suggest that the influencing factors are not mere temperature fluctuation induced by elevation. The contradictory observations may be attributed to the interactive effects of multiple abiotic and biotic factors on microbial community and extracellular enzyme activity, such as temperature, precipitation, vegetation type, soil physical properties, soil pH and nutrient status (Chang et al., 2016; Siles et al., 2017; He X. et al., 2020; Klimek et al., 2020). Disentangling the complex underlying mechanisms driving soil microbial community and extracellular enzyme activity along the elevational gradient is of vital importance to assess and predict the responses of soil microbial community structure and function to climate change (Cui et al., 2016).

The ecological process of the eastern Qinghai-Tibetan Plateau is of particular importance in regulating climate and ensuring ecological security of China (Liu et al., 2020). The geomorphology of this area is characterized by high mountains and deep valleys with a large span of elevation (Cui et al., 2021). Therefore, the area provides an ideal platform for exploring the elevational patterns of soil microbial community and activity, which is helpful to predict the responses of soil function to climate change (He Q. et al., 2020). Several studies addressing the elevational patterns of soil microbial community and extracellular enzyme activity in this area have been reported, but they put focus on either soil microbial community or extracellular enzyme activity (Cao et al., 2021; Cui et al., 2021). The simultaneous understanding of the variations of soil microbial community and extracellular enzyme activity remains lacking and is urgently needed as the area is very sensitive to climate change (Fan et al., 2021). Furthermore, a previous study pointed out a microbial adaption strategy that microorganism would invest more resources in maintaining metabolism relative to growth yield to cope with suboptimal conditions (Malik et al., 2020). Whether soil microorganisms would vary their strategy between metabolism for acquiring resources and biomass synthesis along the elevational gradient is still unclear.

Here, we evaluated the effects of elevation on soil microbial biomass and composition using phospholipid fatty acid (PLFA) analysis, and on extracellular enzyme activities associated with C-, N- and P-acquisition on the eastern Qinghai-Tibetan Plateau. The objectives of the work were to characterize the changes in soil microbial community and extracellular enzyme activities along an elevation gradient and to identify the possible drivers of elevational variation in soil microbial community, and extracellular enzyme activities by investigating their important correlations with climatic factors and edaphic variables. We hypothesized that (i) soil microbial biomass and extracellular enzyme activities would decline markedly at high elevation due to the combined effects of low temperature and edaphic variables that covary with elevation; (ii) soil extracellular enzyme stoichiometry would vary, and C and N/P limitations for microbial metabolism increased along the elevational gradient.

## Materials and methods

### Study site

The study was conducted in Wolong Nature Reserve (102°52′–103°24′E, 30°45′–31°25′N), Wenchuan County, Sichuan Province, southwest of China. It has a temperate subhumid climate with the average annual temperature of 8.5°C and the average annual rainfall of about 890 mm. The landform of the area is characterized by alpine and gorge with elevation ranging from 1,150 to 6,250 m above sea level (a.s.l.). The vegetation distribution has an evident vertical vegetation belt spectrum, with a gradual transition from shrubland, deciduous broad-leaved forest, mixed conifer-broadleaf forest, subalpine dark coniferous forest, shrub-meadow to alpine meadow along the elevational gradient (Chen et al., 2019).

Our experiment was established in coniferous and broadleaved mixed forest (CBF), dark coniferous forest (DCF), subalpine dwarf forest (SDF) and alpine shrubland (AS) locating at elevations of about 2,820, 3,160, 3,420, and 4,280 m a.s.l., respectively. This range of elevation gradient was selected to ensure the soils of different vegetation communities developed from the same parent material, i.e., weathered killas (Liu et al., 2016). The soils were classified as Luvisols according to the WRB classification (IUSS Working Group, 2015). The mean annual temperature at 2820, 3160, 3,420, and 4,280 m a.s.l. was 5.77, 4.21, 3.01, and −0.94°C, respectively, which was calculated using a temperature lapse rate of 0.46°C per 100 m based on three climate stations at 2,800, 3,200, and 3,500 m a.s.l. (Wang et al., 2018). The dominant plant species composition of each elevation is summarized in Table 1.

**Table 1:** Dominant plant species composition at different elevations in Wolong Nature Reserve on the eastern Qinghai-Tibetan Plateau.

Elevation (m a.s.l.)	2,820	3,160	3,420	4,280
Forest type	CBF	DCF	SDF	AS
Dominant trees	*Abies fargesii* var. *faxoniana*; *Betula utilis*; *B. albosinensis*	*A. fargesii* var. *faxoniana*; *Rhododendron* spp.	*Quercus aquifolioides*	*R. nivale*
Understory taxa	*Sorbus koehneana, Cerasus duclouxii*; *Rhododendron* spp.; *Arundinaria faberi*; *Rubus fockeanus*; *Urtica laetevirens*	*Lonicera* spp.; *Arundinaria faberi*; *Ligularia przewalskii*; *Poa nemoralis*	*Pteridium* spp. *Circaeaster agrestis*; *Polygonum* spp.	*Polygonum viviparum*; *Carex* spp.; *Potentilla* spp.

CBF, coniferous and broadleaved mixed forest; DCF, dark coniferous forest; ADF, alpine dwarf forest; AS, alpine shrubland.

### Soil sampling and processing

Five plots were randomly set at each elevation as independent replications (*n* = 5) in July 2018. Each plot was 20 × 20 m except at the elevation of 4,280 m a.s.l., where the plot was 10 × 10 m. In each quadrat, five standard containers (50.46 mm diameter and 50 mm height) were used to collect intact soils at approximately the middle of the topsoil layer (0–10 cm) for measuring soil bulk density (BD) and soil moisture (Liu et al., 2020). Simultaneously, the topsoil (0–10 cm) samples were collected from five locations in each quadrat by a stainless-steel auger of 5 cm diameter after removing surface litter layer. The five soil cores within each quadrat were thoroughly bulked into a composite sample, totaling 20 soil samples (5 replicate samples × 4 elevations) for laboratory analysis, which were then immediately transported to the laboratory using a cooler box with ice packs. The composite samples were sieved through a 2-mm mesh after plant debris and stones were removed. Subsequently, each composite sample was divided into three sub-samples: one of the sub-samples was stored in a refrigerator at −80°C for soil microbial community measurements, one was maintained at 4°C before enzyme assays, and the remaining one was air-dried for determining soil physicochemical properties (Li et al., 2020).

### Physicochemical properties

Intact soil from each standard container was oven-dried at 105°C to constant mass to calculate soil moisture (percentage of soil water in fresh soil) and soil bulk density (SBD, the ratio of soil dry weight to container volume; Xu et al., 2022). Soil pH was determined using soil-water solutions at a ratio of 1:2.5 (*w/v*) by a glass electrode meter. Soil total carbon (TC) and total nitrogen (TN) contents were measured by an elemental analyzer (Elementar Analysen Systeme GmbH, Germany), and total phosphorus (TP) was determined by an inductively coupled plasma-atomic emission spectrometer (iCAP 6300, Thermo Fisher, United States) after digestion with H_2_SO_4_-HClO_4_ (Peng and Wang, 2016). The content of SOM was measured by dichromate oxidation, followed by FeSO_4_ titration (Cui et al., 2021). Dissolved organic carbon (DOC) and total dissolved nitrogen (TDN) contents were determined *via* a TOC analyzer (Multi N/C 3100, Germany) following extraction with 0.5 M K_2_SO_4_ (Qiu et al., 2021). Soil available phosphorus (SAP) was analyzed by the acids (0.05 M HCl-0.025 M H_2_SO_4_) extraction method described by Liu (1996).

### Phospholipid fatty acid analysis

Phospholipid fatty acid (PLFA) analysis was used to assess microbial biomass and community composition. The PLFA contents were determined using the method described by Bossio and Scow (1998). Briefly, lipids were extracted from freeze-dried soil sample (8 g) using a mixture of chloroform, methanol and phosphate (1:2:0.8 *v*/*v*/*v*), and then the solutions were shaken for 2 h and centrifuged for 10 min. The lipids were split into natural lipids, glycolipids and phospholipids by eluting with CHCl_3_, acetone and methanol, respectively. Subsequently phospholipids were methylated with a mild-alkali methanolysis and dried with N_2_. The dried samples were redissolved in hexane containing the internal standard19:0. Samples were analyzed using a Hewlett-Packard gas chromatograph (model 6,890 Series). The individual PLFA was identified with the MIDI Sherlock Microbial Identification System and was quantified based on the internal standard content with units of nmol g^−1^ dry soil (Pollierer et al., 2015). The PLFAs were categorized into diverse taxonomic groups (Yan et al., 2020; Liu et al., 2021), i.e., bacteria [including gram-positive (i13:0, i14:0, i15:0, a15:0, i16:0, a16:0, i17:0, a17:0, i18:0 and i19:0), gram-negative bacteria (14,1ω5, 16,1ω7, 18,1ω7, 18,1ω5, cy17:0, and cy19:0) and general bacteria (15:0 and 17:0)], fungi (18,2ω6,9 and 18,1ω9), and arbuscular mycorrhizal fungi (16,1ω5). The PLFAs ratios of gram-positive/gram-negative bacteria (abbreviated G+/G− ratio) and fungi/bacteria (abbreviated F/B ratio) were calculated to investigate elevational variations in soil microbial community composition.

### Extracellular enzyme activity assays

The potential activities of four extracellular enzymes, namely β-glucosidase (BG), β-*N*-acetylglucosaminidase (NAG), leucine aminopeptidase (LAP), and acid phosphatase (AP) were determined according to a modified and widely used fluorometric protocol (Jing et al., 2020; Jian et al., 2021), with BG, NAG + LAP, and AP indicating C-, N-, and P-acquiring enzymes, respectively (Sinsabaugh et al., 2008). Briefly, soil slurry was prepared by a blender for 1 min with fresh soil (1.25 g) by adding 125 ml of 50 mM sodium acetate buffer (Wang et al., 2020) with a similar pH to that of the soil sample (Zhou et al., 2020; Jian et al., 2021). Soil slurry (800 μl) and 200 μl of 200 μM substrate solution (*viz*. L-leucine-7-amido-methylcoumarin for LAP, and 4-methylumblliferyl for the other three enzymes) were simultaneously pipetted into each well of 96-well microplate with eight replicates per soil sample (Sinsabaugh et al., 2008; Jian et al., 2021). Meanwhile, the wells of quench standards, reference standards, negative controls and blanks were set up for the fluorometric assays as outlined by Wang et al. (2020). The incubation of the prepared solutions was taken in the dark at the temperature of 25°C for 3 h (Li et al., 2021), and 10 μl of sodium hydroxide solution (50 μM) was immediately injected into each well at the end of incubation to terminate the enzymatic reaction (Wang et al., 2020). Fluorescence was quantified using a microplate reader (SpectraMax i3x, Molecular Devices, CA, USA) with an excitation and emission wavelength being 365 nm and 450 nm, respectively (Jian et al., 2021). Enzyme activity was presented as absolute activity in a unit of nmol g^−1^ dry soil h^−1^. Furthermore, specific enzyme activities were normalized by SOM, expressing as nmol g^−1^ SOM h^−1^ (Cui et al., 2021).

Soil enzymatic stoichiometry was calculated as the ratio of C-, N-, and P-acquisition enzymes after ln-transformation (Sinsabaugh et al., 2008; Zhang et al., 2022). Soil microbial metabolic limitation was quantified by vector length and angle analysis based on enzymatic stoichiometry (Sinsabaugh et al., 2009; Li et al., 2020). Vector length represents C limitation, with greater values indicating larger C limitations. Vector angle represents N or P limitation with an angle >45° denoting P limitation and < 45° denoting N limitation (Li et al., 2020).

### Statistical analysis

The variables were tested for normality by the Kolmogorov–Smirnov test, and homogeneity of the variances were checked by the Levene’s test prior to analyses. One-way analysis of variance (ANOVA) and Tukey’s HSD test were used to assess the statistical significance of elevation on soil physiochemical properties, microbial biomass of different functional groups, microbial community composition (F/B and G+/G− ratios), extracellular enzyme activities and stoichiometric ratios, and microbial metabolic limitations (vector angle and length). Linear regression models were used to determine the relations among enzyme activities. The above statistical analyses were conducted with SPSS 22.0 (IBM, United States). The relationships between soil microbial metabolic limitations and MAT, soil microbial community and edaphic variables across the elevations were determined by Pearson correlation analysis using the R package ggcorrplot (Kassambara, 2022). Principal component analysis (PCA) was performed to examine the overall differences in soil microbial community and extracellular enzyme activities among the elevations using the Vegan package in R (Oksanen et al., 2022). The hierarchical partitioning method was used to detect the individual contribution of MAT and edaphic variables to soil microbial community and extracellular enzyme activities using the rdacca.hp. package with low positive values or negative values indicating minor effects (Lai et al., 2022). Furthermore, partial least squares path modeling (PLS-PM) was performed to reveal the indirect and direct effects of MAT, total nutrients and available nutrients on soil microbial community and extracellular enzyme activities using the *innerplot* function in the plspm package (Sanchez et al., 2015). The figures were plotted using SigmaPlot 14.0 (Systat Software Inc. USA) and R (version 4.1.1).

## Results

### Soil physiochemical characteristics

The measured soil physiochemical properties showed different variability along the elevational gradient (Table 2). Soil TC and TN contents, and C:P and N:P ratios decreased and then increased with increasing elevation, showing the lowest values at the mid-elevation (3,420 m). Soil TP and TDN contents generally showed upward trends along the elevational gradient with no significant differences among the elevations of 2,820, 3,160, and 3,420 m. SAP content at 2,820 and 3,420 m was significantly higher than that at other two elevations, whereas the ratios of DOC to SAP and TDN to SAP exhibited contrast trends with significantly higher values at 3,160 and 4,280 m. The DOC:TDN ratio at 3,160 m was significantly higher compared with 4,280 m (*p* < 0.05). However, soil pH, SBD, C:P ratio and DOC content did not vary significantly among the elevations (*p* > 0.05, Table 2).

**Table 2:** Soil physiochemical properties along the elevational gradient.

Parameters	Elevation (m a.s.l.)				One-way ANOVA	
	2,820	3,160	3,420	4,280	*F*	*p*
pH	5.73 ± 0.53	4.65 ± 0.13	5.26 ± 0.10	5.05 ± 0.42	1.68	0.211
SBD (g cm^−3^)	0.76 ± 0.07	0.69 ± 0.05	0.79 ± 0.06	0.50 ± 0.12	2.73	0.079
SM	37.50 ± 1.83b	38.96 ± 2.14b	37.49 ± 3.82b	60.62 ± 6.62a	**7.75**	**0.002**
Total nutrients						
TC (g kg^−1^)	89.04 ± 15.02b	78.43 ± 3.63bc	47.83 ± 2.57c	241.39 ± 12.83a	**73.19**	**<0.001**
TN (g kg^−1^)	5.78 ± 0.59b	5.69 ± 0.39b	3.33 ± 0.18c	17.21 ± 0.77a	**85.87**	**<0.001**
TP (g kg^−1^)	0.57 ± 0.05b	0.69 ± 0.05b	0.82 ± 0.06b	1.39 ± 0.09a	**31.53**	**<0.001**
C:N ratio	15.74 ± 3.00	13.86 ± 0.40	14.40 ± 0.46	14.01 ± 0.28	0.31	0.817
C:P ratio	171.75 ± 48.85a	115.47 ± 3.56ab	59.19 ± 3.83b	175.87 ± 11.65a	**4.74**	**0.015**
N:P ratio	10.39 ± 1.31ab	8.33 ± 0.18b	4.10 ± 0.17c	12.52 ± 0.63a	**23.89**	**<0.001**
Available nutrients						
DOC (mg kg^−1^)	354.74 ± 38.35	452.28 ± 78.32	317.82 ± 39.54	459.25 ± 29.52	1.99	0.156
TDN (mg kg^−1^)	99.68 ± 10.34b	107.39 ± 13.30b	102.14 ± 13.39b	202.94 ± 16.31a	**13.74**	**<0.001**
SAP (g kg^−1^)	46.91 ± 5.75a	18.80 ± 1.78b	46.51 ± 4.71a	14.85 ± 7.32b	**17.38**	**<0.001**
DOC:TDN	3.65 ± 0.40ab	4.11 ± 0.31a	3.26 ± 0.48ab	2.28 ± 0.08b	**4.91**	**0.013**
DOC:SAP	7.98 ± 1.19b	25.32 ± 4.62a	7.14 ± 1.05b	38.59 ± 9.95a	**14.12**	**<0.001**
TDN:SAP	2.40 ± 0.60c	5.98 ± 0.89b	2.29 ± 0.42c	16.97 ± 4.37a	**19.54**	**<0.001**

Data are mean ± standard error. Significant effects of elevation on the soil physiochemical properties are presented in bold. Different letters in the same row indicate significant difference (*p* < 0.05) among elevations. SBD, soil bulk density; SM, soil moisture; TC, total carbon; TN, total nitrogen; TP, total phosphorus; DOC, dissolved organic C; TDN, total dissolved N; SAP, available P.

### Soil microbial community

**Figure 1:**
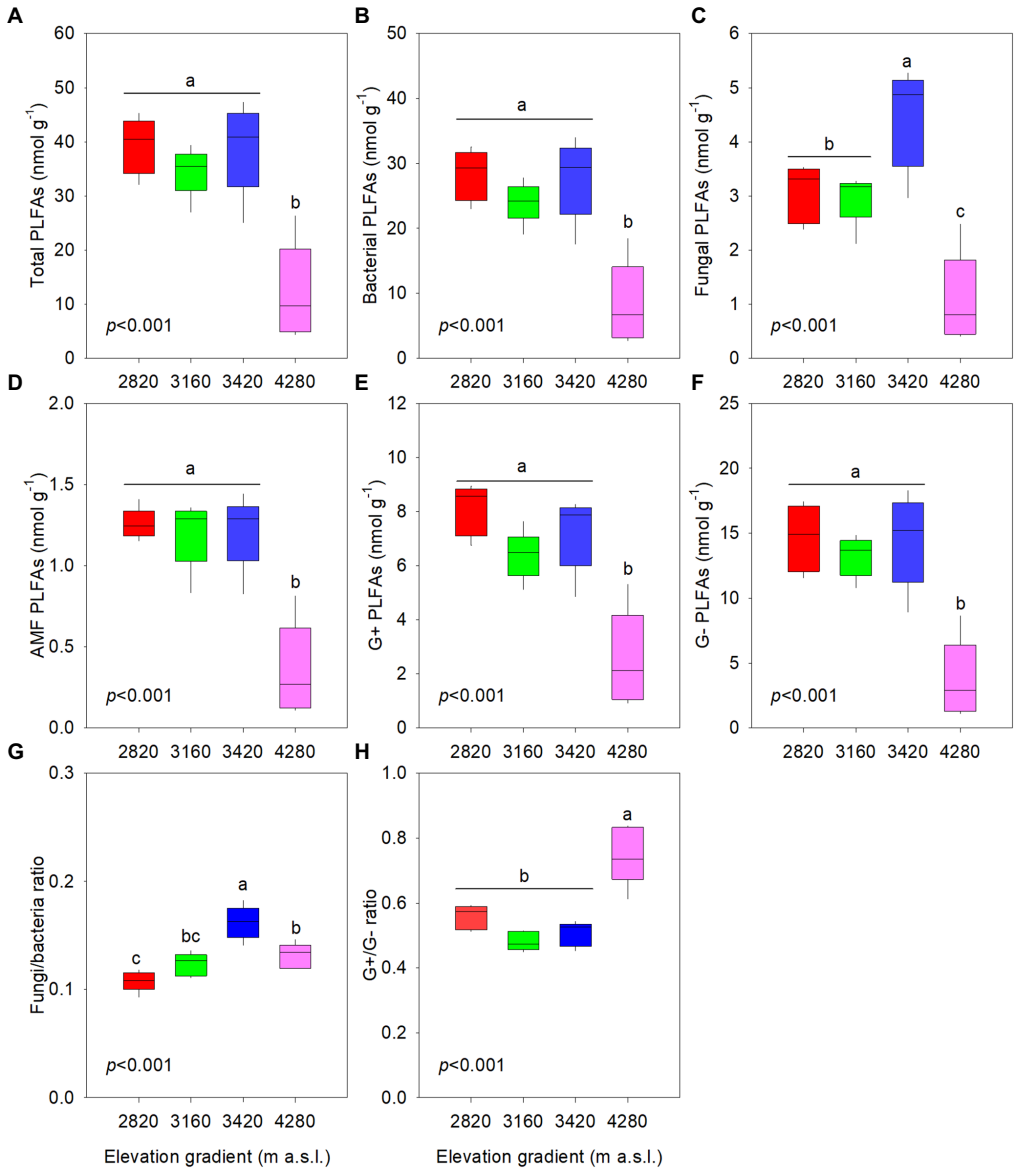
Variations in biomarker contents of soil phospholipid fatty acids (PLFAs) for total microbial biomass **(A)** and different functional groups **(B–F)**, and indicators of soil microbial community **(G–H)** along the elevational gradient. Different letters in each panel indicate significant difference (*p* < 0.05) among elevations. Boxplots represent the minimum, first quartile, median, third quartile and maximum in the data set. AMF, arbuscular mycorrhizal fungi; G+, Gram-positive bacteria; G−, Gram-negative bacteria; F/B, ratio of fungal to bacterial PLFAs; G+/G−, ratio of gram-positive bacterial to gram-negative bacterial PLFAs.

Elevation had significant effects on total PLFAs content and PLFAs contents of different functional groups (*p* < 0.001, Figure 1). Total PLFAs content ranged between 39.30 and 11.97 nmol g^−1^ along the elevational gradient, which was dominated by bacterial PLFAs content ranging from 28.25 to 8.22 nmol g^−1^. The PLFAs content of AMF was lower than that of other functional groups. Consistently, total PLFAs, bacterial (including G+ and G-) and AMF PLFAs contents at 4,280 m were significantly lower than those at other elevations. However, they were not significantly different among the elevations of 2,820, 3,160, and 3,420 m. Fungal PLFAs content was significantly higher at 3,420 m and significantly lower at 4,280 m than that at the other two elevations.

The PFLAs ratios varied significantly with elevation (*p* < 0.05, Figure 1). The F/B ratio generally showed a unimodal pattern along the elevational gradient, exhibiting the highest values at 3,420 m. The G+:G− ratio was significantly highest at 4,280 m and was not significantly different among the other three elevations.

### Soil extracellular enzyme activity and stoichiometry

**Figure 2:**
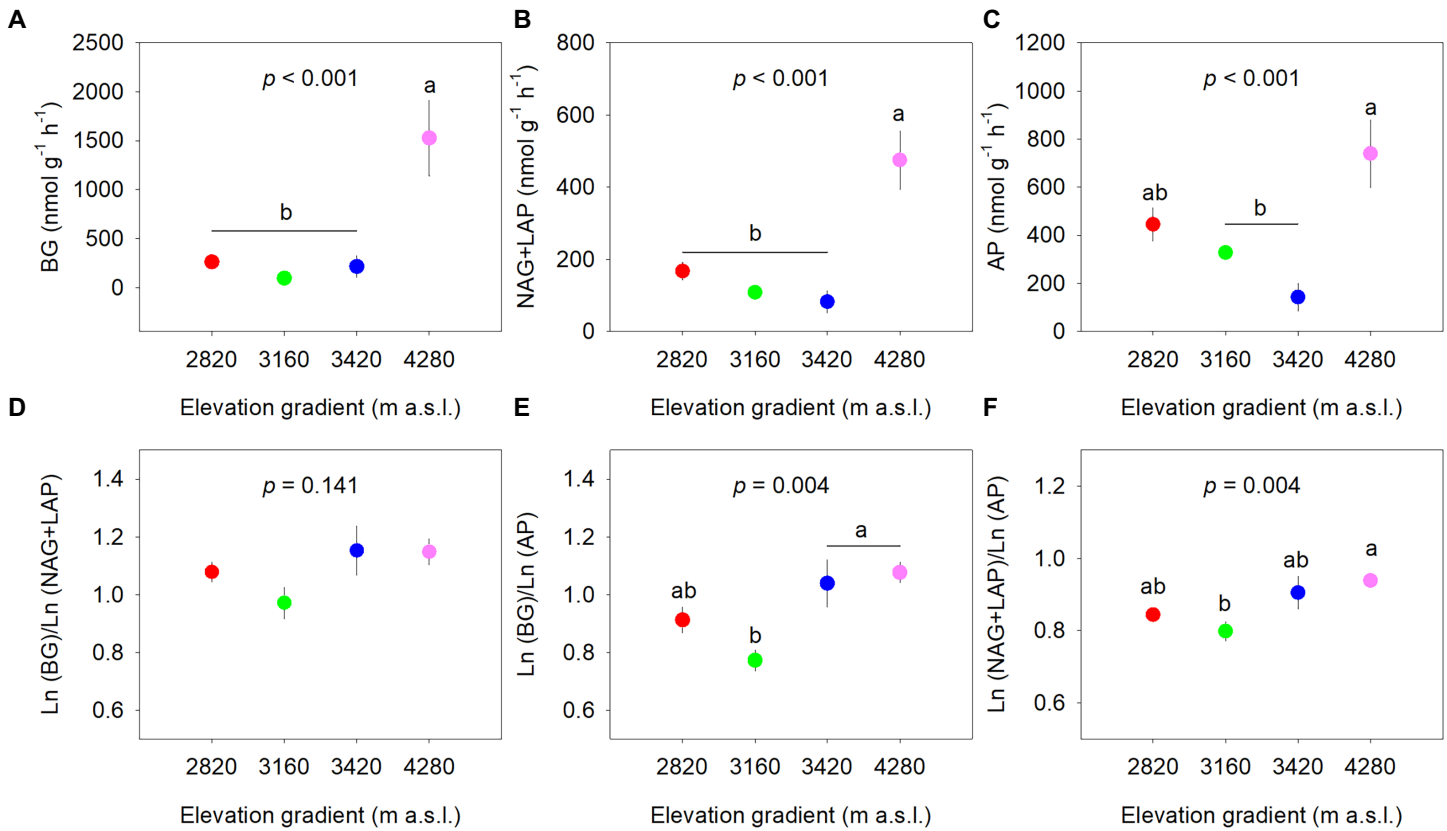
Elevational patterns of soil extracellular enzyme activity normalized by soil **(A–C)** and soil enzymatic acquisition ratio **(D–F)**. Data are expressed as the mean ± standard error. Different letters indicate significant difference (*p* < 0.05) among elevations. BG, β-glucosidase; NAG, β-N-acetylglucosaminidase; LAP, leucine aminopeptidase; AP, acid phosphatase.

The extracellular enzyme activities differed significantly along the elevational gradient (*p* < 0.001, Figures 2A–C). BG and NAG+LAP activities were the highest at 4,280 m (*p* < 0.05) and were not different among other elevations (*p* > 0.05). AP activity at 4,280 m was significantly higher than that at 3,160 and 3,420 m, whereas AP activity at 2,820 m did not differ significantly from other elevations. The specific enzyme activities normalized by SOM were not significantly different among the elevations (Supplementary Figure 1).

The soil enzymatic acquisition ratios generally showed increasing trends along the elevational gradient with significant elevational effects on enzymatic C:P and N:P acquisition ratios, but not on enzymatic C:N acquisition ratio (Figure 2). The enzymatic C:P ratio at 3,420 m and 4,280 m was significantly higher than that at 3,160 m (Figure 2B). The enzymatic N:P ratio was higher at 4,280 m and lower at 3,160 m (Figure 2C). There were significant positive correlations between ln(BG), ln(NAG+LAP), and ln(AP). The relationships between ln(BG) and ln(NAG+LAP), and between ln (NAG+LAP) and ln(AP) seemed to deviate from 1:1, while that between ln(BG) and ln(AP) appeared close to 1:1 (Supplementary Figure 2).

### Soil microbial metabolic limitation

**Figure 3:**
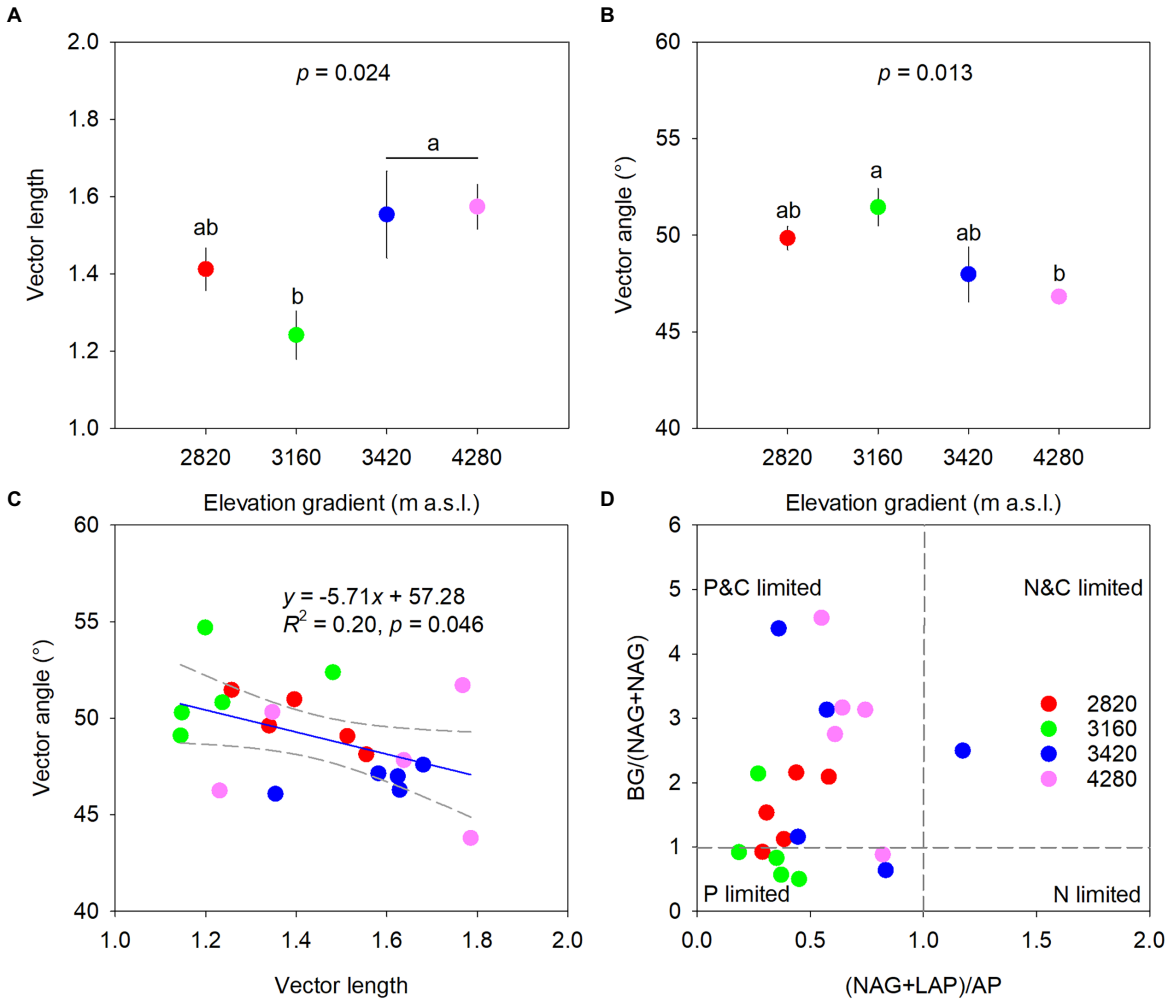
Variations of vector length **(A)** and vector angle **(B)**, and the relationships between vector length and vector angle **(C)** and between BG:(NAG+LAP) and (NAG+LAP):AP **(D)** along the elevational gradient to identify potential resource limitations of soil microbes. Data in the upper two panels are mean ± standard error, and different letters represent significances among elevations at a level of *p* < 0.05. The blue line indicates the linear regression and regression bands of two gray dashed lines represent 95% confidence intervals in the panel **(C)**.

Elevation had a significant effect on microbial metabolic limitations (*p* < 0.05, Figure 3). Specifically, vector length was higher at 4,280 m and 3,420 m and lower at 3160 m with values being 1.57, 1.55, and 1.24, respectively (Figure 3A). Vector angles were greater than 45°, and the elevation of 3,160 m (51.46°) had significantly higher vector angles than the elevation of 4,280 m (46.82°). However, vector angles at 2,820 and 3,420 m did not differ significantly from those at 3,160 and 4,280 m, respectively (Figure 3B). Vector angle had a significant negative correlation with vector length (*R*^2^ = 0.20, *p* = 0.046, Figure 3C). In addition, most samples had a (NAG+LAP): AP ratio of less than 1 across the elevations (Figure 3D).

### Key factor affecting soil microbial community and extracellular enzyme activity

**Figure 4:**
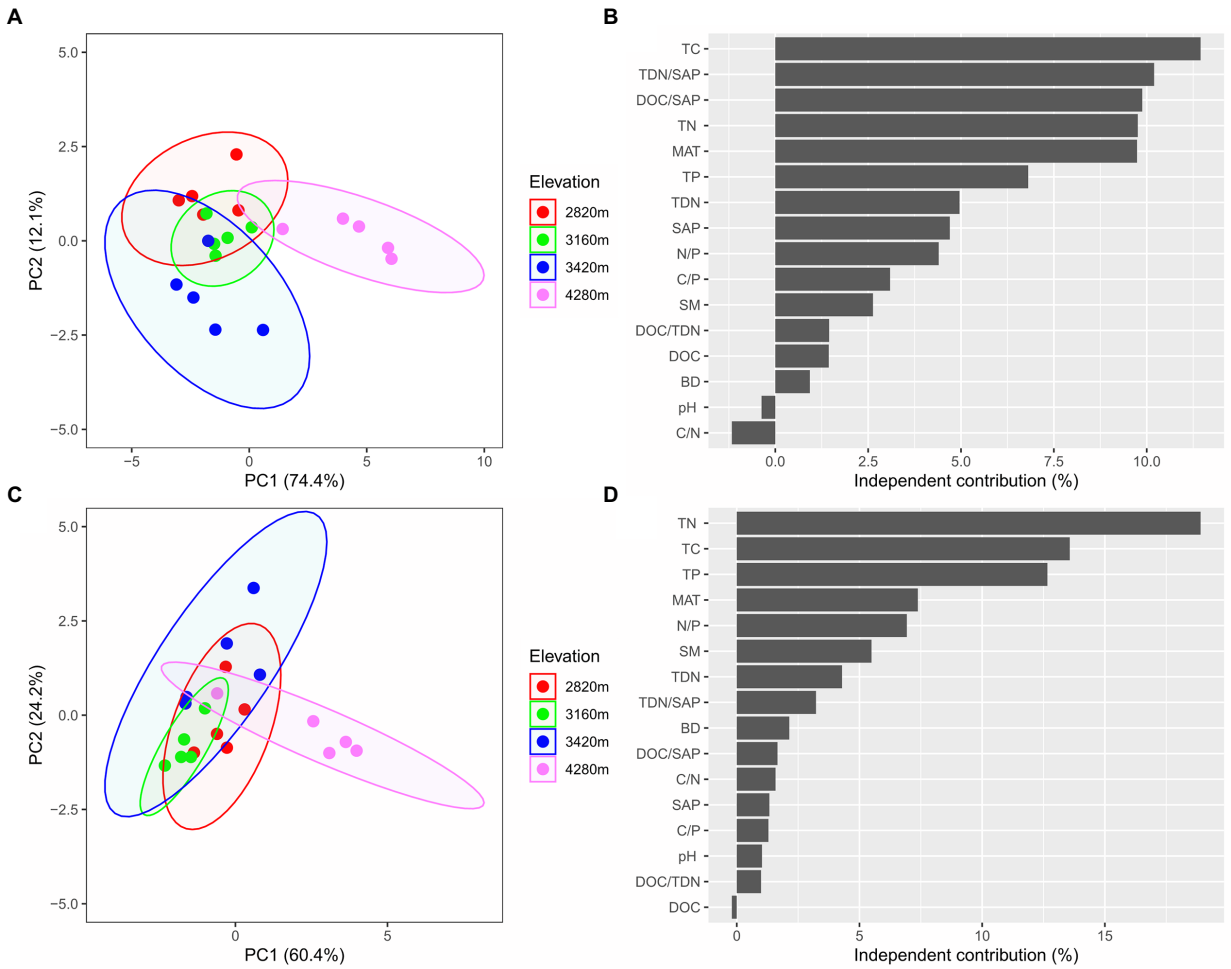
The biplots of the principal component (PCA) analysis based on microbial PLFAs **(A)** and based on extracellular enzyme activities **(C)** reveal the changes in soil microbial community composition and extracellular enzyme activity characteristics along the elevational gradient, respectively, and the independent contribution of explanatory variables (including MAT and edaphic factors) to the variations in soil microbial community composition **(B)** and extracellular enzyme activities **(D)** by the hierarchical partitioning analysis.

The PCA showed that elevation had significant effects on soil microbial community composition as well as extracellular enzyme activity characteristics (Figures 4A,C). The first two principal components explained approximately 86.5% of the total variation in soil microbial PLFAs (of which PC1 explained 74.4% and PC2 explained 12.1%), and approximately 84.6% of the total variation in soil enzyme activity characteristics with PC1 and PC2 explaining 60.4 and 24.2%, respectively.

**Figure 5:**
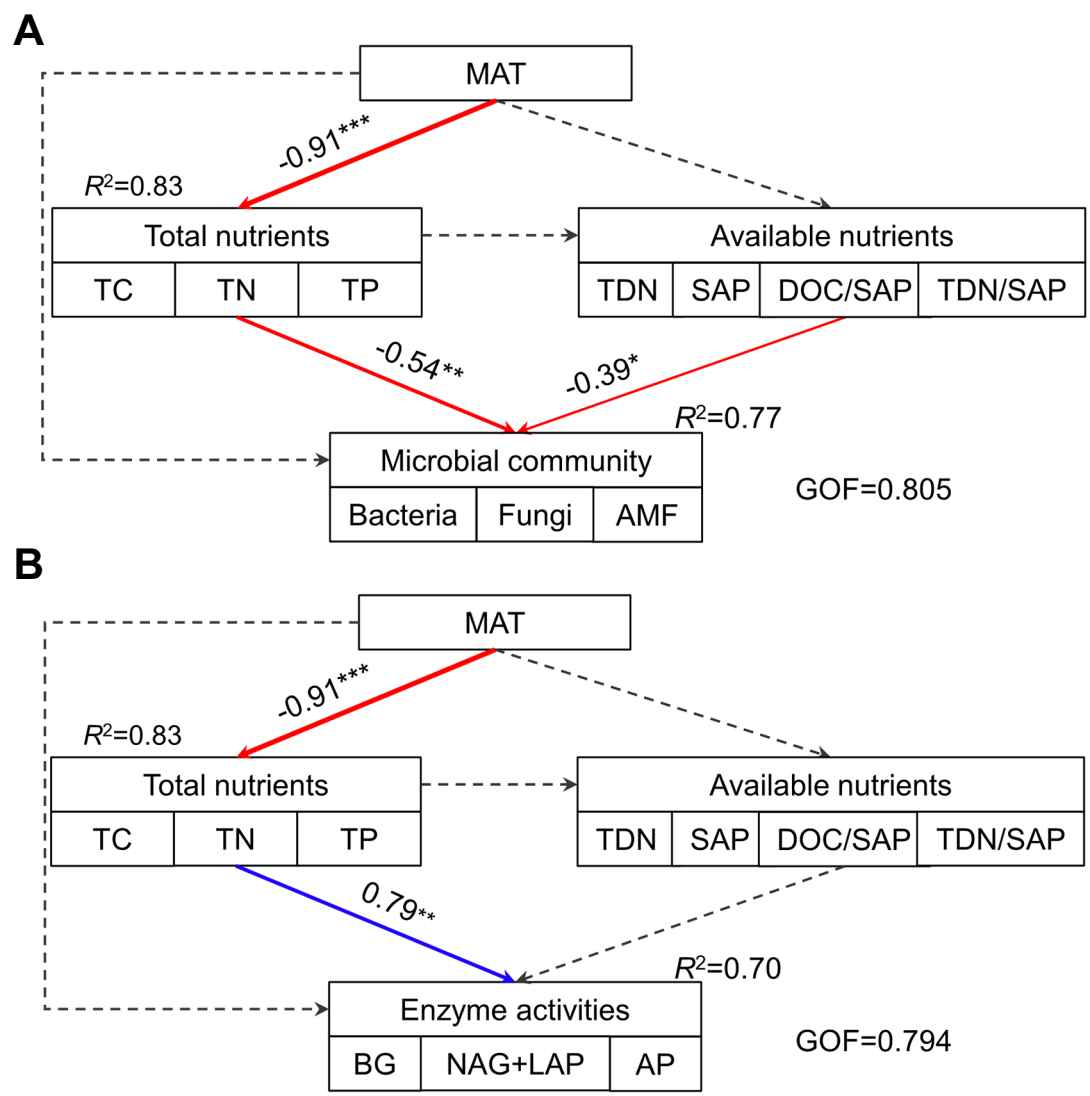
The path model describing the direct and indirect effects of MAT, soil total nutrients and available nutrients on soil microbial community **(A)** and extracellular enzyme activities **(B)**. Continuous arrows indicate significant relationships with blue representing positive effects and red representing negative effects, while dashed arrows indicate nonsignificant relationships. The width of arrows characterizes the strength of path coefficients. *R*^2^ values indicate the proportion of variance explained. GOF, goodness of fit; MAT, mean annual temperature; TC, total carbon; TN, total nitrogen; TP, total phosphorus; TDN, total dissolved N; SAP, available P; DOC/SAP, the ratio of DOC to SAP; TDN/SAP, the ratio of TDN to SAP.

The hierarchical partitioning analysis showed that TC was the most important factor influencing soil microbial community composition along the elevational gradient with the independent contribution being 11.5%, followed by TDN/SAP, DOC/SAP, TN, MAT, and TP (Figure 4B). However, soil total nutrients had the most important effect on the elevational pattern of soil extracellular enzyme activities, followed by MAT (Figure 4D). The PLS-PM indicated that MAT affected soil microbial biomass and extracellular enzyme activities indirectly *via* influencing soil total nutrients (Figure 5). Soil total nutrients and available nutrients displayed significant negative effects on soil microbial community, whereas soil total nutrients exhibited a significant positive correlation with extracellular enzyme activities.

**Figure 6:**
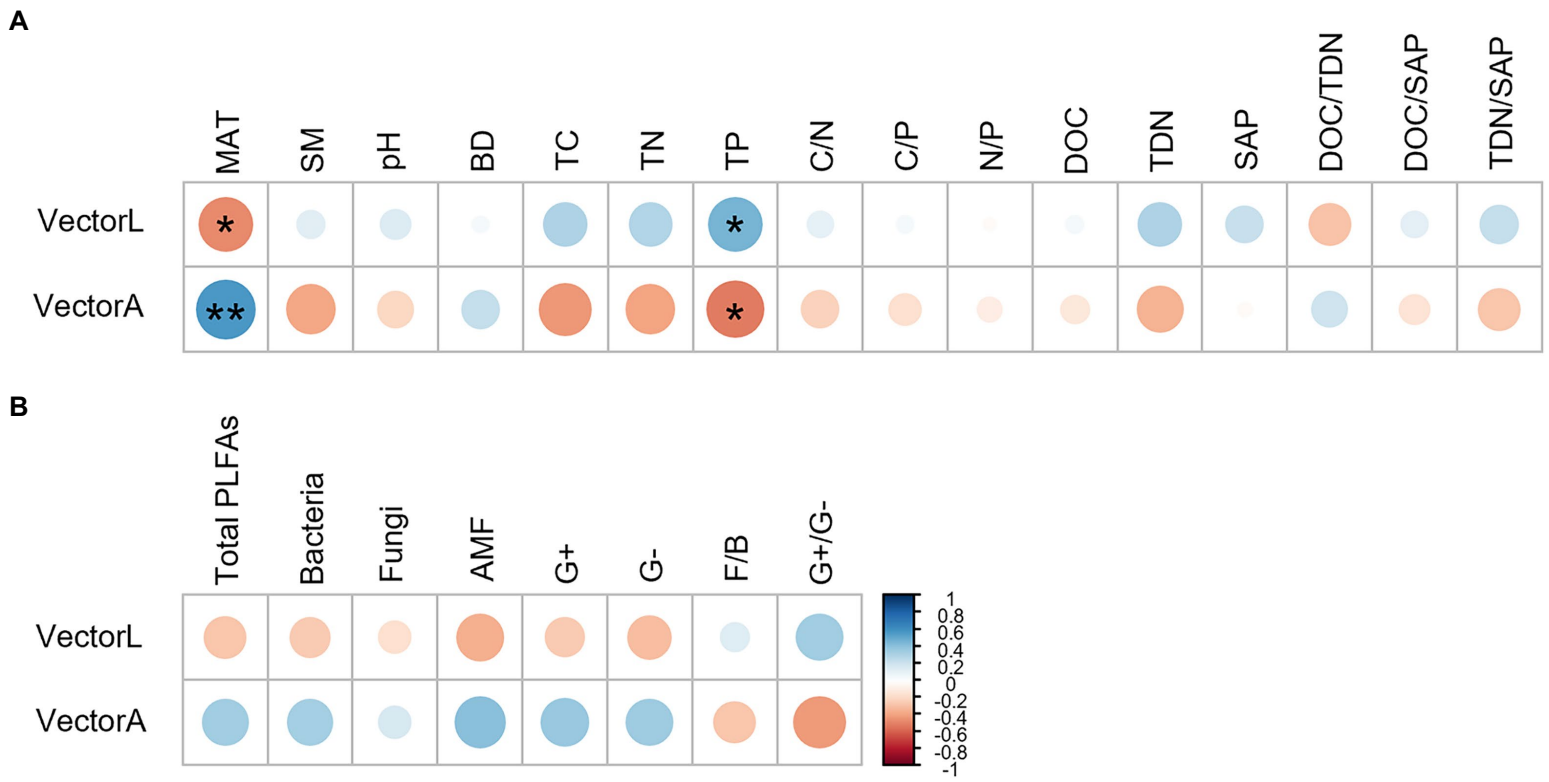
Pearson correlation analysis showing the relationship between microbial resource limitation (the vector length and angle) and explanatory variables, including MAT, soil physiochemical factors **(A)** and microbial community composition **(B)**. **p < 0.01, *p < 0.05.

The correlation analysis showed that the vector length and angle were correlated significantly with MAT and TP (Figure 6). MAT had a negative correlation with the vector length and a positive correlation with the vector angle. TP correlated positively with the vector length and negatively with the vector angle.

## Discussion

### Drivers of soil microbial community along the elevational gradient

We found the lowest microbial biomass (indicated by PLFAs) at the highest elevation (except fungi) (Figure 1), which partly supported the first hypothesis. Similar patterns were also observed in the Austrian Central Alps (Margesin et al., 2009) and Mount Segrila (Xu et al., 2014). Nonetheless, Liu et al. (2019) documented an increasing and then decreasing tendency of microbial biomass with elevation in Changbai Mountain of China, and Siles et al. (2016) reported a higher microbial biomass at higher elevations in the Italian Alps. These diverse results supported the finding of no consistent elevational pattern for microbial biomass globally (He X. et al., 2020). The inconsistency might be owed to the fact that microbial biomass is governed by a variety of factors such as climatic conditions, edaphic parameters, and vegetation type, any of which except temperature could vary inconsistently with elevation, thus leading to diverse results (Xu et al., 2014; Hu et al., 2016). A potential mechanism might be attributed to the range of climatic conditions, as contrasting responses of microbial biomass to temperature and precipitation were observed in different climate zones (Xu et al., 2013). He X. et al. (2020) also revealed that the decrease in temperature induced by elevation enhanced microbial biomass in the sub-/tropical zones, but not in other climate zones. Temperature could regulate microbial metabolism directly, and indirectly by altering C and nutrient availability (Peng et al., 2022). High temperature could either stimulate microbial biomass by enhancing plant productivity and SOM input or reduce microbial biomass by increasing SOM decomposition and nutrient availability (Chen et al., 2016). In addition, although the low temperature at high elevations might favor nutrient accumulation, its limitation of mineralization would also inhibit nutrient availability and microbial biomass (Hu et al., 2016). This to some extent highlighted the important influence of elevation range selection on the results (Zhang et al., 2013). An alternative explanation might be the specific vegetation types differing in productivity, diversity, litter input and soil characteristics along elevational gradients, in turn resulting in contrasting elevational patterns of microbial biomass among studies (He X. et al., 2020; Massaccesi et al., 2020). For example, a case study revealed the positive effect of plant diversity on microbial biomass with elevation (Ren et al., 2018), whereas the elevational patterns for plant biodiversity did not obtain a universal pattern (Guo et al., 2013). In addition, Klimek et al. (2020) observed that plant diversity had a positive effect on only some specific PLFAs, and other PLFAs were more related to soil properties, which could also explain the contradictory results of previous studies addressing microbial biomass with elevation.

A shifted soil microbial community composition along the elevational gradient was detected (Figures 1, 4) and might be driven by an integrated effect of abiotic and biotic factors (Xu et al., 2015; Kotas et al., 2018). We detected different elevational patterns in fungal biomass versus bacterial biomass (Figures 1B,C), showing the highest fungi/bacteria ratio at 3,420 m (Figure 1G). This supported, to some extent, that microbial community composition varied across the elevations, and on the other hand indicated their different responses to elevation changes (Margesin et al., 2009). Compared to bacteria, fungi are more efficient in decomposing recalcitrant compounds (Hackl et al., 2005). The litter of *Q. aquifolioides* might contain high amounts of phenolic compounds such as tannin (Escandón et al., 2021), and the degradation of complex compounds favored propagation of fungi at 3,420 m (Wagai et al., 2011), thus causing the results of high fungal biomass (Figure 1C) and fungi/bacteria ratio (Figure 1G) at 3,420 m.

In addition, the G+/G− ratio varied significantly along the elevational gradient, with the maximum at 4,280 m (Figure 1H). This possibly indicated that bacteria at this elevation were somehow more severely resource-limited than at other elevations since G+ bacteria appeared to be more resistant to harsh and resource-constrained conditions than G- bacteria (Bai et al., 2017; Lei et al., 2017) Interestingly, we found that soil total nutrient contents (TC, TN and TP) were significantly higher at 4,280 m (Table 2) with their levels being comparable to those of shrub tundra dominated with *Rhododendron chrysanthum* in Changbai Mountain of China (Jin et al., 2019). The alpine shrubland at 4,280 m with high coverage (95%) might have large amounts of roots with a short lifespan (Liu et al., 2018) and low decomposition induced by low temperature, probably contributing to its higher total nutrient levels. Soil total nutrient contents, especially TC had an important explanation for soil microbial community (Figure 4B), however, they were correlated with soil microbial biomass negatively (Figure 5A). The result contradicted the finding of a positive correlation between total nutrients and soil microbial biomass obtained from numerous studies (Whitaker et al., 2014; He X. et al., 2020). The primary reasons for the negative relation in our study might be related to temperature-mediated microbial metabolism and nutrient decomposition.

The low temperature at high elevation might increase physiological stress by changing osmotic pressure and decreasing the ability to obtain available nutrients (Ren et al., 2018, 2021; Nottingham et al., 2019), which in turn reduced microbial biomass. Moreover, it could lead to declined mineralization and decomposition, thus limiting nutrient availability and favoring nutrient accumulation at high elevation (Sveinbjörnsson et al., 1995; Shedayi et al., 2016). However, soil microbial biomass synthesis and population proliferation require nutrient uptake from degrading organic substrates (Xu et al., 2015), implying a significant role of nutrient availability in microbial biomass and composition (Hu et al., 2016). The low nutrient availability induced by temperature might potentially limit microbial biomass at high elevation, thus resulting in a negative relationship between total nutrient accumulation and microbial biomass across the elevations (Figure 5A). In this way, we observed a strong explanation for microbial biomass by available nutrients (Figure 5A), and SAP was positively correlated with total microbial biomass as well as each subgroup. Meanwhile, the DOC/SAP and TDN/SAP ratios had negative impacts on microbial community with high independent contributions (Figure 4B), further suggesting an important role of SAP in affecting microbial community as their higher ratios could reveal the relatively stronger deficiency of SAP according to stochiometric theory (Hartman and Richardson, 2013). Overall, these results might illustrate a strong control of substrate availability on soil microbial biomass and community (Zhang et al., 2013).

### Drivers of soil extracellular enzyme activity along the elevational gradient

We found a significant effect of elevation on soil extracellular enzyme activities with the highest values at 4,280 m (Figure 2). The result partly contradicted the first hypothesis and was inconsistent with previous studies that demonstrated a decreasing trend along an elevation covered 1,308–2,600 m (Ren et al., 2021) and no significant change along an elevation covered 130–640 m (Truong et al., 2019). The discrepancy might be caused by the different vegetation cover and climatic regimes under specific elevational gradients in these studies (Ren et al., 2021) because extracellular enzyme activities are very sensitive to environmental signals (Jiang et al., 2009; Sinsabaugh et al., 2009). A similar trend that greater extracellular enzyme activities occurred at higher elevations was also observed from 3,200 to 4,000 m in a nearby site (Fan et al., 2021), which might further support the above views.

There is ample evidence that soil extracellular enzyme activities are linked closely to a suite of biotic and abiotic factors (Xu et al., 2017; Ivashchenko et al., 2021). We observed similar trends for the extracellular enzyme activities involved in C-, N- and P-acquisition along the elevational gradient (Figure 2), suggesting the same influencing factors for them and a tight coupling cycle of C, N, and P (Waring et al., 2014; Fan et al., 2021). Extracellular enzyme activities in our study were tightly linked to soil total nutrient contents (i.e., TC, TN, and TP), confirming the view that soil total nutrients play a key role in mediating elevational patterns of extracellular enzyme activities (Chang et al., 2016; Truong et al., 2019; Cao et al., 2021). In addition, a non-significant elevational effect on specific activities per SOM (Supplementary Figure 1) indicated that the variations in extracellular enzyme activities along the elevational gradient were highly in line with SOM accumulation (Ivashchenko et al., 2021).

MAT might play an important influence on extracellular enzyme activities (Figure 4D) with an indirect effect through total nutrients (Figure 5B). The relationship between extracellular enzyme activities and MAT was negative (Figure 5B), which was also found in local and regional scale studies (Peng and Wang, 2016; Xu et al., 2017). In contrast, Ren et al. (2021) observed a positive relationship between enzyme activities and MAT, whereas Cao et al. (2021) found enzyme activities were independent of temperature across an elevation gradient and a similar result was reported from a global synthesis (Sinsabaugh et al., 2008). The contradicted relationships implied that MAT might indirectly affect enzyme activities, thus supporting our result (Figure 5B). The low temperature suppresses the decomposition, thus stimulating microbial enzyme secretion to obtain nutrients. Another possible reason for the indirect effect of MAT was that the relatively low temperature was not conducive to the rapid turnover of enzymes (He Q. et al., 2020), further contributing to high enzyme activities at 4,280 m.

Sinsabaugh et al. (2008) suggested that the ratios of enzymes involved in C-, N- and P- acquisition converged on 1:1:1 in global ecosystems. However, the important influence of total nutrients on enzyme activities in this study might indicate that soil enzymatic stoichiometry was resource dependent rather than homeostatic (Peng and Wang, 2016). Indeed, the ln-transformed enzymatic C:N:P acquisition ratios deviated slightly from the 1:1:1 ratio (Supplementary Figure 2), and similar results were also found in previous studies on different spatial scales (Zhou et al., 2020; Wu et al., 2021). Likewise, elevation had significant effects on enzymatic stoichiometries except for the enzymatic C:N acquisition ratio (Figure 2). Soil C-:P-acquiring and N-:P- acquiring enzyme ratios were relatively low at low elevations (i.e., 2,820 and 3,160 m), indicating that microorganisms tended to invest more P-acquisition enzyme and experienced strong P limitation at these elevations. Such a conclusion could also be confirmed by the elevational pattern of the vector angle (Figure 3B). The decreased microbial P limitation along the elevational gradient partly contradicted our second hypothesis and was also found in subalpine soils (Liao et al., 2021) and tropical forest soils (Nottingham et al., 2015).

The relationship between BG:(NAG+LAP) ratio and (NAG+LAP):AP ratio revealed that microbial metabolism was simultaneously constrained by C and P (Figure 3D). In contrast to the elevational pattern of microbial P limitation, microbial C limitation generally increased with elevation, which partly supported our second hypothesis. The vector length and angle were significantly correlated with TP content across the elevation gradient, highlighting the important role of TP in regulating microbial metabolism. We found that TP increased with increasing elevation (Table 2). A major driver appeared to be the decreasing degree of soil weathering with elevation since soil P was mainly sourced from weathered soils (Helfenstein et al., 2018). Furthermore, high productivity at low elevation might strongly deplete primary minerals and intensify the competition for P between plants and microorganisms, thereby increasing microbial P limitation (Nottingham et al., 2015). Meanwhile, high productivity at low elevation could stimulate rhizodeposition and C source, consequently alleviating microbial C limitation (Cui et al., 2021). Interestingly, we observed no clear elevational trend for DOC, and the maximum TC at the highest elevation was accompanied by severe microbial C limitation. We speculated that it might be attributed to the large investment of energy for enzyme synthesis (Figure 2) and the high metabolic quotient (respiration per unit biomass) at the high elevation (Feng et al., 2021).

### Ecological implications and limitations

We found that microbial biomass and enzyme activities showed contrasting elevational patterns with the minimum microbial biomass and the maximum enzyme activities at the highest elevation (Figures 1, 2). This supported the contention of the inconsistent relationships between soil microbial biomass and enzyme activities (Li Y. et al., 2018), and reflected their different drivers along the elevational gradient. A little confusion was that SAP might limit soil microbial biomass synthesis at high elevation which experienced a milder P limitation of microbial metabolism compared to low elevation. It was reasonable because microorganisms would give priority to the investment in acquiring enzymes to obtain resource in the case of resource limitation (Malik et al., 2020; Shao et al., 2021).

Notably, the greatest enzyme activities at the highest elevation did not mean the maximum catalytic rate *in situ* due to the low temperature. It might indicate more accumulated enzymes or higher temperature sensitivity of enzymes because the method for the measurement of enzyme activities determined the potential activities under the constant temperature and was widely used in previous studies (Liu et al., 2020; Wang et al., 2020; Jian et al., 2021). High enzyme activities and strong microbial C limitation in soils of the highest elevation might imply that it’s SOC decomposition and turnover would be more strongly stimulated under climate warming than other elevations. We therefore speculated that the effects of climate warming on soil C stock were probably more drastic at the highest elevation, especially as it was observed to have the highest TC content (Table 2). Overall, these results might have important implications for understanding soil function along the elevational gradient in this area under climate change.

In addition, we found little variation in soil microbial biomass and enzyme activities among 2,820, 3,160, and 3,420 m where the vegetation type was mixed conifer-broadleaf forest, dark coniferous forest and alpine dwarf forest, respectively. The finding was similar to the study conducted in Gongga Mountain of China, which found the stair-step pattern of soil bacterial diversity along an elevation gradient and primarily driven by vegetation type (Li J. et al., 2018). In this study, we paid more attention to the effects of soil properties like some other studies (Zhang et al., 2013), as vegetation might play an indirect effect through soil properties (Ushio et al., 2008). Future studies addressing the effects of vegetation on soil microbial community and enzyme activity in this area need to strengthen. Overall, the stair-step pattern highlighted that the conversion of shrubland to forest due to the upward timberline migration under climate warming might have a considerable effect on soil nutrient cycling and function *via* altering soil microbial community and enzyme activities.

## Conclusion

Soil microbial biomass and enzyme activities generally showed contrasting responses to elevation with the lowest microbial biomass and the greatest enzyme activities at the highest elevation, possibly implying a shifted microbial strategy between resource acquisition and population construction to adapt to the harsh conditions. Soil microbial metabolism was mainly limited by C and P, generally exhibiting an increasing C limitation but a decreasing P limitation with elevation. Soil total nutrients and available nutrients, especially P availability were the main determinants of microbial community, whereas total nutrients mainly explained the variation of enzyme activities along the elevational gradient. Microbial metabolic limitations were related to MAT and TP across the elevations. Overall, these results highlighted the vital role of soil P in affecting the elevations variations of soil microbial community and metabolism. This will help to understand and predict the response of belowground community and function to climate change on the eastern Qinghai-Tibetan Plateau.

## Data availability statement

The original contributions presented in the study are included in the article/Supplementary material, further inquiries can be directed to the corresponding author.

## Author contributions

SL and ZS conceived the study and designed the experiment. SL, GX, HC, MZ, XC, MC, and JC conducted the field experiment. SL, MZ, XC, MC, and JC performed the laboratory work. SL analyzed the data and wrote the manuscript. QF and ZS reviewed and edited the manuscript. All authors contributed to the article and approved the submitted version.

## Funding

This work was supported by the Fundamental Research Funds of Chinese Academy of Forestry (CAFYBB2018ZA003, CAFYBB2021ZA002-2, and CAFYBB2017MA008), the Open Project Program of Ecological Restoration and Conservation on Forest and Wetland Key Laboratory of Sichuan Province (2020KFKT02) and the National Key Research and Development Program (2021YFD2200405 and 2016YFC0502104-02).

## Conflict of interest

The authors declare that the research was conducted in the absence of any commercial or financial relationships that could be construed as a potential conflict of interest.

## Publisher’s note

All claims expressed in this article are solely those of the authors and do not necessarily represent those of their affiliated organizations, or those of the publisher, the editors and the reviewers. Any product that may be evaluated in this article, or claim that may be made by its manufacturer, is not guaranteed or endorsed by the publisher.
