# Supplementary material for: Contrasting responses of soil microbial biomass and extracellular enzyme activity along an elevation gradient on the eastern Qinghai-Tibetan Plateau
Source: Front Microbiol. 2023 Jan 18;14:974316. doi: 10.3389/fmicb.2023.974316 (PMC9889656; doi:10.3389/fmicb.2023.974316)
Supplement: Supplementary file 1 [file Data_Sheet_1.docx]

Supplementary Material

**Supplementary Figure 1.** Elevational patterns of specific soil enzyme activity normalized by soil organic matter (SOM). Data are expressed as the mean ± standard error. Different letters indicate significant difference (p < 0.05) among elevations. BG, β-glucosidase; NAG, β-N-acetylglucosaminidase; LAP, leucine aminopeptidase; AP, acid phosphatase.

**Supplementary Figure 2.** The relationships between extracellular enzyme activities after the ln-transformation across the elevations. Red, green, blue, and pink represent 2820 m, 3160 m, 3420 m, and 4280 m, respectively. BG, β-glucosidase; NAG, β-N-acetylglucosaminidase; LAP, leucine aminopeptidase; AP, acid phosphatase.
